# Association between Food Preferences and Food Habits in a Polish Adolescents’ COVID-19 Experience (PLACE-19) Study

**DOI:** 10.3390/nu13093003

**Published:** 2021-08-28

**Authors:** Dominika Skolmowska, Dominika Głąbska, Dominika Guzek

**Affiliations:** 1 Department of Dietetics, Institute of Human Nutrition Sciences, Warsaw University of Life Sciences (SGGW-WULS), 159C Nowoursynowska Street, 02-776 Warsaw, Poland; dominika_skolmowska@sggw.edu.pl; 2 Department of Food Market and Consumer Research, Institute of Human Nutrition Sciences, Warsaw University of Life Sciences (SGGW-WULS), 159C Nowoursynowska Street, 02-776 Warsaw, Poland; dominika_guzek@sggw.edu.pl

**Keywords:** food preferences, food habits, Food Preference Questionnaire (FPQ), Adolescents’ Food Habits Checklist (AFHC), adolescents, national study, population-based study, PLACE-19 Study

## Abstract

Food preferences are among the most influential factors of food habits in the vulnerable period of adolescence; in addition, gender-dependent differences in food preferences are also observed. The aim of the present study was to analyze differences in food habits between individuals stratified based on their food preferences in a population-based sample of adolescents aged 15–20. The study was conducted within the Polish Adolescents’ COVID-19 Experience (PLACE-19) Study population in a group of 2419 secondary school students who were randomly chosen to participate in the study using a random quota sampling procedure. The food preferences were determined on the basis of a validated Food Preference Questionnaire (FPQ) (which enables assessing preference of vegetables, fruit, meat/fish, dairy, snacks, and starches), whereas food habits were determined on the basis of the Adolescents’ Food Habits Checklist (AFHC) (which enables assessing food purchase, preparation, and consumption habits). The analysis involved three homogenous clusters (‘low-preferring’, ‘hedonists’, and ‘high-preferring’), which were identified using the k-means algorithm. It was found that for a number of the assessed food purchase, preparation, and consumption habits, there were statistically significant differences between the ‘low-preferring’, ‘hedonists’, and ‘high-preferring’ clusters. Within food purchase habits, the food preference influenced frequency of buying pastries/cakes and frequency of eating takeaway meals for all the respondents, while for female respondents, it influenced also choice of desserts in restaurants, and for male respondents, it influenced choosing a low-fat lunch away from home (*p* < 0.05). Within food preparation habits, the food preference influenced the fat content in desserts at home, the frequency of eating at least one serving of vegetables/salad with an evening meal, the frequency of spreading butter/margarine on bread thinly, and the frequency of having cream on desserts for all the respondents; meanwhile, for female respondents, it also influenced the frequency of avoiding fried foods and the frequency of including chocolate/biscuits in their packed lunch (*p* < 0.05). Within food consumption habits, the food preference influenced the frequency of eating a dessert/pudding, eating at least one serving of fruit a day, eating at least one serving of vegetables/salad a day, avoiding sausages/burgers, trying to ensure they eat plenty of fruit and vegetables, and frequency of choosing fruit as a snack for all the respondents; meanwhile, for male respondents, it also influenced the frequency of eating sweet snacks and eating at least three servings of fruit most days (*p* < 0.05). Taking into account that some improper food habits may be typical for the specific clusters, there is an urgent need to analyze and address them for the purposes of public health and to bear in mind that some of those habits are gender-dependent.

## 1. Introduction

Adolescence is known to be a critical period of development in the lifespan [1]. In this time, the developmental, physical, and social changes that occur play an important role in establishing eating behaviors, while adolescents’ food habits significantly affect both their current and future health [2]. Adolescence is also a period of susceptibility on a number of factors that influence the food habits [3]. One of them is family environment, as it is indicated that positive family interactions may promote healthy food habits [4]. Peers are also considered to affect adolescents’ eating habits, and according to the systematic review of Ragelienė and Grønhøj, this influence is often rather negative than positive [5]. Another determinant is media influence, as it has been shown that the media environment shapes food-related attitudes, knowledge, preferences, and practices [6]. Moreover, exposure to child-targeted fast food television advertising has been linked with fast food intake in a group of pre-school children [7]. Apart from parents, peers, and mass media, the food preferences are among the most influential factors of food habits in adolescents [8,9].

Food preferences are defined as the evaluative attitudes that are expressed toward food and include how much people like or dislike the specific food products [10]. Their development begins at conception, and infants display characteristic preference of taste, with sweet and umami showing positive response, while sour and bitter show a negative response [11]. It is also revealed that food preferences are developed through consistent exposure to food; therefore, the availability and accessibility of certain food products will be of importance while determining whether children will display healthy or not healthy food preferences [12]. Studies indicate that the food preferences influence different aspects associated with individuals’ health, such as weight status [13] and nutritional status [14]. In spite of the fact that the food preferences continue to change throughout the life course while influenced by biological, environmental, and social factors, they remain key determinants of the diet quality [15]. Food preferences have been also linked to gender [16]. In the study of Caine-Bish and Scheule [17] conducted in a group of school-age children and adolescents, it was stated that boys showed higher preference toward meat, fish, and poultry than girls, while girls preferred vegetables and fruits over boys.

Studies suggest that the specific food and taste preferences may predispose either to healthy or unhealthy food habits. In the study of Diószegi et al. [18] conducted in Hungarian adults, it was stated that higher preference for bitter flavor was associated with higher vegetable consumption, as well as higher sweet food preference was associated with lower consumption of fruits. In the study of Sluik et al. [19] conducted within the Consortium on Health and Ageing: Network of Cohorts in Europe and the United States (CHANCES) project, the preferences influenced intake of fruits and vegetables and resultant general quality of diet. A study by Lanfer et al. [20] indicated that children with high preference of fat had a significantly higher frequency of consumption of fried fish, fried potatoes, or fried meat than those with low preference of fat.

As indicated by the United Nations Children’s Fund (UNICEF), middle childhood and adolescence give opportunity for growth, psychosocial development, and establishing lifelong food habits; therefore, optimal diet and proper nutrition are crucial in this period [21]. As a result, it is a matter of great concern to understand the determinants underlying food habits in order to manage adolescents’ diet for preventing improper dietary habits and diet-related diseases [9]. Taking this into account, the aim of the present study was to analyze differences of food habits between individuals stratified based on their food preferences in a population-based sample of adolescents aged 15–20.

## 2. Materials and Methods

### 2.1. Ethical Statement

The PLACE-19 Study received approval of the Ethics Committee of the Institute of Human Nutrition Sciences of the Warsaw University of Life Sciences, to be conducted in a population-based sample of adolescents [22,23,24,25,26]. Both the participants and their parents/legal guardians provided informed consents to participate in the study, and all the procedures were carried out in compliance with the Declaration of Helsinki.

### 2.2. Studied Population

The PLACE-19 Study consisted of two phases, which assessed different aspects of the life of Polish secondary school students (aged 15–20) during the COVID-19 pandemic. The first phase focused on their hygienic and personal protective behaviors [27,28,29], while the second phase analyzed their dietary habits [22,23,24,25,26]. The presented study, similar to the previous one [25], is based on the clustering of the studied sample based on the food preferences assessed while using the Food Preference Questionnaire (FPQ) by Smith et al. [30]. In Poland, the current Net Enrollment Rate (NER) for this level of education is 89.38% [31].

The study was conducted using a random quota sampling procedure with quotas for voivodeships and counties, as it was described in the previous study [23]. The Headmasters of each randomly selected secondary school received an invitation to participate in the study, and if they agreed, they forwarded a link to the electronic version of the questionnaire to the students. Participation in the study was voluntary.

The gathered sample consisting of 2448 secondary school students was considered to be a population-based one, as participants were recruited from all regions of Poland, and the proportional share of respondents from different regions of Poland was maintained.

The inclusion criteria were set as follows: being a student of a randomly selected secondary school, age of 15–20 years, and providing informed consent to participate, both by students and their parents/legal guardians. The exclusion criteria were set as follows: answers in the questionnaire that were considered unreliable, and answers in the questionnaire that were missing. Finally, the sample of 2419 was used for this analysis.

### 2.3. Applied Questionnaires

The present study was conducted in the period of April–May 2020. In this period, strict lockdown was introduced to minimize the transmission of the virus, similarly as in the other European countries in the same period [32]. The lockdown included closure of borders for noncitizens, cancellation of mass events and temporary closure of museums, art galleries and theatres, while restaurants could have only provided food for delivery and takeaway, in shopping malls only groceries, pharmacies, and laundries remained open, as well as citizens were recommended not to leave their homes unless necessary [33]. In relation to the decision of the Polish Ministry of National Education [34], all classes and lectures at schools and the universities were suspended and students were in the system of remote learning during the course of the study. Therefore, the applied questionnaire was distributed among participants using the Computer-Assisted Web Interview (CAWI) method by the use of Google Forms, while no sensitive or personal data were collected, expect for sex, age, and attended school, so a single respondent could not be identified while completing the survey, and filling out the questionnaire was anonymous.

The food preferences of the respondents were assessed based on the FPQ by Smith et al. [30]. The FPQ is a reliable, validated, self-reported tool to be used in the group of children and adolescents. The FPQ contains a list of 62 various food products, and the respondents determine how much on average they like the specific food product with the possible answers as follows: (1) dislike a lot, (2) dislike a little, (3) neither like nor dislike, (4) like a little, (5) like a lot (for any item they have ever tried, independently from the actual consumption), as well as (6) not applicable (for any item they do not know, or do not remember ever having tried) [33]. The FPQ enables determining the preferences of vegetables (questionnaire contains 18 food products within this food category), fruit (7 items), meat/fish (12 items), dairy (10 items), snacks (9 items), and starches (6 items), which may be obtained by adding the single food preference item scores within each food category and dividing this sum by the number of items [35].

The food habits were determined based on Adolescents’ Food Habits Checklist (AFHC) by Johnson et al. [36], which was validated in a group of adolescents. Within this study, the Polish version of the AFHC was applied, which was translated from English to Polish according to the WHO recommendations, as described in the previous study [23]. The AFHC includes questions related to purchasing, preparing, and consuming different kinds of food products (defined as healthy and unhealthy food habits). Respondents were asked to declare if they present described habits or not, and in case of some questions, they had also a possibility to declare the ‘not applicable’ option to be chosen if they do not eat some groups of products at all. Habits associated with purchasing, preparing, and consuming were analyzed separately as within the previous study [24].

### 2.4. Statistical Analysis

To verify the normality of distribution, the Shapiro–Wilk test was applied. For clustering, the k-means algorithm was used with Euclidean distance to distinguish homogenous clusters of respondents according to their preferences, and the optimal number of clusters was verified using the Elbow method.

To compare groups, the chi^2^ test was performed.

The statistical significance was set for *p* ≤ 0.05. The statistical analysis was performed using Statistica version 13.3 (StatSoft Inc., Tulsa, OK, USA), Statgraphics Plus for Windows 5.1 (Statgraphics Technologies Inc., The Plains, VA, USA) and JASP version 0.14.0.0 (JASP Team, 2020).

## 3. Results

The food preferences assessed while using the FPQ in the adolescent group within the PLACE-19 Study are presented in Table 1. The k-means algorithm was applied to define the main clusters. Three homogenous clusters were identified. The first cluster (named ‘low-preferring’) was characterized by low preference for all food categories. The second cluster (named ’hedonists’) gathered respondents declaring high preference for snacks and fruit but not for other products. The third cluster (named ‘high-preferring’) was characterized by declared high preference for all food categories.

The demographic characteristics within the clusters stratified based on the preferences assessed while using the FPQ in the adolescent group within the PLACE-19 Study are presented in Table 2. It was stated that the highest share of males was attributed to the ‘high-preferring’ cluster (41.0% of cluster) compared with the ‘low-preferring’ (35.6%) and ‘hedonists’ clusters (32.6%) (*p* < 0.001). At the same time, the highest share of older respondents was attributed to the ‘high-preferring’ cluster (52.9% and 8.1% of cluster for age groups of 17–18 and 19–20 years, respectively) compared with the ‘low-preferring’ (48.9% and 6.3%) and ‘hedonists’ clusters (49.3% and 4.5%) (*p* < 0.001).

The declared food purchase habits in the clusters stratified based on the preferences in the adolescents within PLACE-19 Study are presented in Table 3. Respondents from the ‘low-preferring’ cluster more often than respondents from other clusters declared choosing a low-fat lunch away from home (*p* = 0.001), choosing a low-fat crisp brand (*p* = 0.001), and choosing the healthiest dessert in a restaurant (*p* = 0.007). Moreover, respondents from the ‘hedonists’ cluster more often than respondents from other clusters declared buying pastries or cakes (*p* = 0.001) and rarely eating takeaway meals (*p* < 0.001). At the same time, respondents from the ‘high-preferring’ cluster less often than respondents from other clusters declared choosing a diet drink (*p* = 0.004).

The declared food preparation habits in the clusters stratified based on the preferences in the adolescents within the PLACE-19 Study are presented in Table 4. Respondents from the ‘low-preferring’ cluster more often than respondents from other clusters declared usually avoiding eating fried foods (*p* < 0.001), choosing at home dessert low in fat (*p* < 0.001), as well as less often than respondents from other clusters declared including chocolate or biscuits into a packed lunch (*p* < 0.001), spreading butter or margarine on a bread thinly (*p* < 0.001), and having cream on dessert (*p* < 0.001). Moreover, respondents from the ‘high-preferring’ cluster more often than respondents from other clusters declared usually eating at least one serving of vegetables with their evening meal (*p* < 0.001).

The declared food consumption habits in the clusters stratified based on the preferences in the adolescents within the PLACE-19 Study are presented in Table 5. Respondents from the ‘low-preferring’ cluster more often than respondents from other clusters declared usually eating a dessert (*p* < 0.001) and eating sweet snacks between meals (*p* = 0.006); in addition, they less often than respondents from other clusters declared eating at least one serving of fruit a day (*p* < 0.001) and at least one serving of vegetables a day (*p* < 0.001), choosing fruit when they have a snack between meals (*p* < 0.001), ensuring they eat plenty of fruit and vegetables (*p* < 0.001), avoiding eating lots of sausages and burgers (*p* < 0.001), and trying to have a healthy diet (*p* = 0.019).

The declared food purchase habits in the clusters stratified based on the preferences in the female adolescent group within the PLACE-19 Study are presented in Table 6. Respondents from the ‘hedonists’ cluster more often declared buying pastries or cakes than the ‘high-preferring’ cluster, while the ‘high-preferring’ cluster did it more often than the ‘low-preferring’ cluster (*p* = 0.030). Moreover, respondents from the ‘high-preferring’ and the ‘hedonists’ clusters more often declared rarely eating takeaway meals than those from the ‘low-preferring’ cluster (*p* = 0.001). At the same time, respondents from the ‘low-preferring’ cluster more often declared choosing the healthiest dessert or pudding in a restaurant (*p* = 0.005), as well as more often declared buying a low-fat crisps brand (*p* = 0.015) than those from the ‘hedonists’ and the ‘high-preferring’ clusters.

The declared food preparation habits in the clusters stratified based on the preferences in the female adolescent group within the PLACE-19 Study are presented in Table 7. Respondents from the ‘low-preferring’ cluster more often declared usually avoiding eating fried foods than the ‘hedonists’ cluster, while the ‘hedonists’ cluster did it more often than those from the ‘high-preferring’ cluster (*p* < 0.001). Respondents from the ‘high-preferring’ cluster less often declared that if they are having a dessert at home, they try to have something low in fat (*p* < 0.001) than the ‘low-preferring’ and the ‘hedonists’ clusters. Moreover, respondents from the ‘high-preferring’ cluster more often declared usually eating at least one serving of vegetables or salad with the evening meal (*p* = 0.004) and usually including some chocolate and/or biscuits to a packed lunch (*p* < 0.001) than the ‘hedonists’ cluster, while the ‘hedonists’ cluster did it more often than the ‘low-preferring’ cluster. However, respondents from the ‘low-preferring’ cluster less often declared that if they put butter or margarine on bread, they usually spread it thinly (*p* = 0.001), and they less often declared having cream on desserts (*p* = 0.001) than the ‘hedonists’ and the ‘high-preferring’ clusters.

The declared food consumption habits in the clusters stratified based on the preferences in the female adolescent group within the PLACE-19 Study are presented in Table 8. Respondents from the ‘low-preferring’ cluster more often declared usually eating a dessert or pudding if there is one available than the ‘hedonists’ cluster, while the ‘hedonists’ cluster did it more often than the ‘high-preferring’ cluster (*p* < 0.001). Respondents from the ‘high-preferring’ cluster more often declared making sure they eat at least one serving of fruit a day (*p* = 0.013), avoiding eating lots of sausages and burgers (*p* < 0.001), making sure they eat at least one serving of vegetables or salad a day (*p* < 0.001) and trying to ensure they eat plenty of fruit and vegetables (*p* < 0.001) than the ‘hedonists’ cluster, while the ‘hedonists’ cluster did it more often than the ‘low-preferring’ cluster. At the same time, respondents from the ‘hedonists’ cluster more often declared that if they have a snack, they often choose fruit (*p* < 0.001) than the ‘high-preferring’ cluster, while the ‘high-preferring’ cluster did it more often than the ‘low-preferring’ cluster.

The declared food purchase habits in the clusters stratified based on the preferences in the male adolescent group within PLACE-19 Study are presented in Table 9. Respondents from the ‘low-preferring’ cluster more often declared that if they are having lunch away from home, they often choose a low-fat option than those belonging to the ‘hedonists’ and the ‘high-preferring’ clusters (*p* = 0.043). Respondents from the ‘hedonists’ cluster more often declared often buying pastries or cakes than those belonging to the ‘low-preferring’ and the ‘high-preferring’ clusters (*p* = 0.029). Moreover, respondents from the ‘low-preferring’ cluster less often declared rarely eating takeaway meals than the ‘hedonists’ and the ‘high-preferring’ clusters (*p* = 0.035).

The declared food preparation habits in the clusters stratified based on the preferences in the male adolescent group within the PLACE-19 Study are presented in Table 10. Respondents from the ‘low-preferring’ cluster more often declared that if they are having a dessert at home, they try to have something low in fat (*p* = 0.001), as well as that they try to keep their overall fat intake down (*p* < 0.001) than those from the ‘hedonists’ and the ‘high-preferring’ clusters. Respondents from the ‘high-preferring’ cluster more often declared usually eating at least one serving of vegetables or salad with the evening meal than the ‘hedonists’ cluster, while the ‘hedonists’ cluster did it more often than the ‘low-preferring’ cluster (*p* < 0.001). However, respondents from the ‘hedonists’ cluster more often declared that if they put butter or margarine on bread, they usually spread it thinly (*p* = 0.001), as well as often having cream on desserts than the ‘high-preferring’ cluster, while the ‘high-preferring’ cluster did it more often than the ‘low-preferring’ cluster (*p* = 0.006).

The declared food consumption habits in the clusters stratified based on the preferences in the male adolescent group within the PLACE-19 Study are presented in Table 11. Respondents from the ‘low-preferring’ cluster more often declared usually eating a dessert or a pudding if there is one available (*p* < 0.001) and often eating sweet snacks between meals (*p* = 0.011) than the ‘hedonists’ cluster, while the ‘hedonists’ cluster did it more often than the ‘high-preferring’ cluster. Moreover, respondents from the ‘hedonists’ cluster more often declared avoiding eating lots of sausages and burgers (*p* < 0.001) than those from the two other clusters. Respondents from the ‘high-preferring’ cluster more often declared making sure they eat at least one serving of fruit a day (*p* = 0.001), making sure they eat at least one serving of vegetables or salad a day (*p* = 0.001), and trying to eat plenty of fruit and vegetables (*p* < 0.001) than the ‘hedonists’ cluster, while the ‘hedonists’ cluster did it more often than the ‘low-preferring’ cluster. Additionally, respondents from the ‘low-preferring’ cluster less often declared choosing fruit when they have a snack between meals (*p* < 0.001) than the ‘hedonists’ and the ‘high-preferring’ clusters. Respondents from the ‘high-preferring’ cluster more often declared eating at least three servings of fruit most days (*p* = 0.038) than respondents from the ‘low-preferring’ and the ‘hedonists’ clusters.

The summary of the comparison of the declared food habits assessed based on the AFHC in the clusters stratified based on the preferences assessed while using the FPQ within the population of the second phase of the PLACE-19 Study for female and male respondents is presented in Supplementary Table S1.

## 4. Discussion

### 4.1. General

Within the present study, the conducted analysis enabled distinguishing three different clusters of adolescents based on their food preferences and determining their food habits. It was found that for a majority of assessed food purchase, preparation, and consumption habits, there were statistically significant differences between the ‘low-preferring’, ‘hedonists’, and ‘high-preferring’ clusters, and that they were gender-dependent. Therefore, there is an urgent need to analyze and address improper food habits and patterns that are typical for the specific clusters for female and male individuals [37].

### 4.2. Problems within ‘Hedonists’ Cluster

In our study, the highest number of adolescents was categorized into the ‘hedonists’ cluster. Adolescents belonging to this cluster declared high preference only for snacks and fruit but not for other products. Such observation is in compliance with other studies, which confirm that younger-aged individuals show a higher preference toward products characterized by a high content of sugar [38]. It corresponds with the fact that in our own study, the ‘hedonists’ cluster more often declared buying pastries or cakes than two other clusters, both in the female and male subgroups. Such behavior may adversely influence their body weight, as in the study of Sobek et al. [39], it was found that in a group of children and adolescents who preferred the high-sweet taste, the frequency of obesity was twice as high as in a group of individuals with the low-sweet taste preference. Regardless of the gender, the ‘hedonists’ cluster along with the ‘high-preferring’ cluster showed also a high consumption of cream, as the majority of the respondents declared that they often have cream on desserts. Such food habit that results from their preference toward sweet taste may be associated with higher intake of energy, fat, and sugar that increases the risk of obesity [40].

However, some beneficial food habits may be also observed within the ‘hedonists’ cluster, as adolescents within this cluster more often declared choosing fruit when they have a snack between meals than others. However, it should be born in mind that the fruit consumption may be treated as a beneficial food habit only if they are consumed in a moderate amount, as an excessive intake of fruit may favor body weight gain [41], while it may be supposed that the ‘hedonists’ may be prone to excessive consumption of fruit due to their increased preference [42]. Moreover, female adolescents belonging to the ‘hedonists’ cluster comparing to other clusters more often declared that if they are having a dessert at home, they try to have something low in fat. Such an observation was not noticed for the male subgroup, which may result from the fact that males pay less attention to fat content in the food products [43], so it should be emphasized that this association may be considered as a gender-dependent difference.

### 4.3. Problems within the ‘High-Preferring’ Cluster

The ‘high-preferring’ cluster gathered adolescents who expressed their preference toward all groups of food products. In the study of Grieger et al. [44], Australian girls were assigned to different clusters based on their consumption, and the cluster named ‘combination’ included adolescents consuming a variety of different food products, both core (cereals, vegetables, fruit, meat) and noncore ones, which were characterized by a significantly higher intake of carbonated sugar drinks, takeaway food, and potatoes than the other clusters. Taking this into account, it may be supposed that this cluster, due to its high preference toward all food products, may be prone to some unhealthy food habits [44]. In the presented own study, the ‘high-preferring’ cluster also showed some negative food habits, as more often than the other clusters, female respondents denied choosing the healthiest dessert or pudding in a restaurant, less often declared avoiding eating fried food, and more often declared usually including some chocolate and/or biscuits to a packed lunch. The above-mentioned association was gender-dependent, as for male respondents, such differences between clusters were not observed, which may indicate that female high-preferring respondents are more prone than male ones to some unhealthy food habits, due to their increased preference for fatty sweet products compared with male [45]. However, for the other products, a higher food preference in male respondents was revealed, as female adolescents within the ‘high-preferring’ cluster more often than the other clusters declared avoiding eating lots of sausages and burgers, while in the male sub-group, the ‘high-preferring’ cluster declared it less often compared to other clusters. It may result from the fact that men generally tend to have high preference toward meat [46], so for the ‘high-preferring’ cluster, this preference may be reflected within their dietary habits. Taking this into account, it may be stated that adolescents belonging to the ‘high-preferring’ cluster may be prone to gender-dependent overconsumption, comparing to the other clusters.

On the other hand, adolescents from the ‘high-preferring’ cluster displayed also some positive food habits, as regardless of the gender, they more often than the other clusters declared usually eating at least one serving of vegetables or salad with the evening meal and/or daily, making sure they eat at least one serving of fruit a day, and trying to ensure they eat plenty of fruit and vegetables. The described habits may be associated with an increased preference of all food products, including healthy ones, which corresponds with the increased consumption of them, as in general, the preference is associated with increased consumption, which is confirmed not only for individuals but is also stated within a household [47].

The positive food habits observed for the ‘high-preferring’ cluster were also gender-dependent. In case of male adolescents from the ‘high-preferring’ cluster, they less often declared buying pastries or cakes, which may result from the fact that men generally tend to have less craving for sweet foods than women [48]. Males belonging to the ‘high-preferring’ cluster also less often declared eating sweet snacks between meals than the other clusters, which may have resulted from the increased size of meals and meal frequency for ‘high-preferring’ male respondents, causing a lower frequency of snacking between meals for male individuals than for female ones, which was observed for students also by other authors [49]. Moreover, within the ‘high-preferring’ cluster, male individuals exclusively declared eating at least three servings of fruit most days more often than the other clusters, which may be explained by the general lower fruit consumption for males than females in various studies [50,51], causing only ‘high-preferring’ males to have increased fruit consumption.

### 4.4. Problems within ‘Low-Preferring’ Cluster

Adolescents from the ‘low-preferring’ cluster had also some health-promoting food habits resulting from the avoiding of different products. Regardless of gender, this cluster more often than ‘hedonists’ denied buying pastries or cakes and denied often having cream on dessert. Some health-promoting food habits may be also considered as gender-dependent. The highest number of male respondents from this cluster declared that if they are having a dessert at home, they try to have something low in fat, while the highest number of female respondents from this cluster declared that they usually avoid eating fried foods, declared that they usually choose the healthiest dessert or pudding in a restaurant, and denied including chocolate and/or biscuits in a packed lunch, comparing to the other clusters. It should be indicated that the described differences may result from the general gender-dependent preferences and intake of food products—higher intake of meat for men [52] and sweets for women [16], which corresponds the fact that only in the case of the ‘low-preferring’ cluster, female respondents declared reduced desserts consumption and choosing the healthiest one.

### 4.5. Future Perspectives

There is growing evidence that the existing food preferences may be gender-dependent [16,53], and in our study, both male and female respondents had some inappropriate food habits, which were dependent on the cluster and gender. In the study of Cooke and Wardle [16] carried out in a group of children and adolescents, it was found that girls showed higher preference toward fruit and vegetables than boys, while boys preferred meat, meat products, as well as sugary and fatty foods more than girls did. Similarly, in the study of Kimura et al. [54], it was revealed that boys were characterized by higher fat preference than girls. However, such specific preferences may change during maturation [11], so older adolescents may be characterized by different preferences than younger children. Moreover, specific preferences and resultant gender-dependent dietary behaviors may contribute to the development of overweight or obesity [20]. Therefore, it is important to introduce different strategies and nutrition education, which should be adjusted to the needs of specific genders and clusters in order to improve children’s and adolescents’ diet quality. Such an approach will enable expanding their awareness, knowledge, and willingness both to taste and eat health-promoting food products and to reduce their negative food habits [55].

In spite of the fact that the presented study provided some novel observations, it should be mentioned that it assessed only the food habits, and no resultant body mass changes were analyzed. As the study was not a longitudinal one, it was not possible to observe the changes of body mass and body composition caused by the changes of food habits during the COVID-19 pandemic. Taking this into account, further studies are needed.

## 5. Conclusions

A conducted analysis enabled distinguishing three different clusters of adolescents based on their food preferences and determining their food habits. Therefore, there is an urgent need to analyze and address improper food habits, which are typical for the specific clusters for the purposes of public health. Moreover, there were also some gender-dependent differences, so it may be suggested that public health actions should be personalized and dedicated for specific cluster and gender.

## Figures and Tables

**Table 1:** The food preferences assessed while using the Food Preference Questionnaire (FPQ) within the population of the second phase of the Polish Adolescents’ COVID-19 Experience (PLACE-19) Study (*n* = 2419).

Variable ^1^	Cluster 1 ‘Low-Preferring’ (*n* = 270)				Cluster 2 ‘Hedonists’ (*n* = 1109)				Cluster 3 ‘High-Preferring’ (*n* = 1040)			
	Mean	SD	M	IQR	Mean	SD	M	IQR	Mean	SD	M	IQR
Vegetable	2.5	0.6	2.5 *	0.8	3.5	0.6	3.6 *	0.7	4.2	0.5	4.2 *	0.7
Fruit	2.8	0.8	3.0 *	0.9	4.3	0.6	4.3 *	0.8	4.7	0.4	4.9 *	0.4
Meat/fish	2.4	0.7	2.3 *	1.0	3.2	0.7	3.3 *	0.8	4.0	0.6	4.0 *	0.8
Diary	2.6	0.6	2.6	0.8	3.4	0.5	3.4 *	0.7	4.2	0.5	4.2 *	0.7
Snacks	2.8	0.7	2.9 *	0.7	4.1	0.6	4.1 *	0.8	4.6	0.5	4.8 *	0.7
Starches	2.8	0.7	2.8 *	0.8	3.9	0.6	4.0 *	0.6	4.5	0.5	4.5 *	0.6

^1^ Scores based on adding the single food preference item scores within each food category and dividing this sum by the number of items with the possible answers as follows: (1) dislike a lot, (2) dislike a little, (3) neither like nor dislike, (4) like a little, (5) like a lot; * distribution different than normal; SD—standard deviation; M—median; IQR—interquartile range.

**Table 2:** The demographic characteristics within the clusters stratified based on the preferences assessed while using the Food Preference Questionnaire (FPQ) within the population of the second phase of the Polish Adolescents’ COVID-19 Experience (PLACE-19) Study (*n* = 2419).

Variable		Cluster 1 ‘Low-Preferring’ (*n* = 270)	Cluster 2 ‘Hedonists’ (*n* = 1109)	Cluster 3 ‘High-Preferring’ (*n* = 1040)	*p*-Value
Sex	Female	174 (64.4%)	747 (67.4%)	614 (59.0%)	<0.001
	Male	96 (35.6%)	362 (32.6%)	426 (41.0%)	
Age (years)	15–16	121 (44.8%)	512 (46.2%)	406 (39.0%)	<0.001
	17–18	132 (48.9%)	547 (49.3%)	550 (52.9%)	
	19–20	17 (6.3%)	50 (4.5%)	84 (8.1%)	

**Table 3:** The declared food purchase habits assessed based on the Adolescents’ Food Habits Checklist (AFHC) in the clusters stratified based on the preferences assessed while using the Food Preference Questionnaire (FPQ) within the population of the second phase of the Polish Adolescents’ COVID-19 Experience (PLACE-19) Study (*n* = 2419).

Declared Food Purchase Habits Based on AFHC *		Cluster 1 ‘Low-Preferring’ (*n* = 270)	Cluster 2 ‘Hedonists’ (*n* = 1109)	Cluster 3 ‘High-Preferring’ (*n* = 1040)	*p* **
If I am having lunch away from home, I often choose a low-fat option	True	79 (29.3%)	263 (23.7%)	253 (24.3%)	0.001
	False	83 (30.7%)	357 (32.2%)	405 (38.9%)	
	Not applicable	108 (40.0%)	489 (44.1%)	382 (26.7%)	
If I am buying crisps, I often choose a low-fat brand	True	57 (21.1%)	190 (18.5%)	175 (18.2%)	0.001
	False	130 (48.1%)	634 (54.5%)	641 (60.3%)	
	Not applicable	83 (30.7%)	285 (27.0%)	224 (21.5%)	
I often buy pastries or cakes	True	200 (74.1%)	904 (81.5%)	788 (75.8%)	0.001
	False	70 (25.9%)	205 (18.5%)	252 (24.2%)	
I rarely eat takeaway meals	True	196 (72.6%)	926 (83.5%)	864 (83.1%)	<0.001
	False	74 (27.4%)	183 (16.5%)	176 (16.9%)	
When I am buying a soft drink, I usually choose a diet drink	True	113 (41.9%)	457 (41.2%)	361 (34.7%)	0.004
	False	157 (58.1%)	652 (58.8%)	679 (65.3%)	
If I am having a dessert or pudding in a restaurant, I usually choose the healthiest one	True	52 (19.2%)	165 (14.9%)	152 (14.6%)	0.007
	False	113 (41.8%)	477 (43.0%)	514 (49.4%)	
	Not applicable	105 (38.9%)	467 (42.1%)	374 (36.0%)	

* AFHC—Adolescents’ Food Habits Checklist; ** chi^2^.

**Table 4:** The declared food preparation habits assessed based on the Adolescents’ Food Habits Checklist (AFHC) in the clusters stratified based on the preferences assessed while using the Food Preference Questionnaire (FPQ) within the population of the second phase of the Polish Adolescents’ COVID-19 Experience (PLACE-19) Study (*n* = 2419).

Declared Food Preparation Habits Based on AFHC *		Cluster 1 ‘Low-Preferring’ (*n* = 270)	Cluster 2 ‘Hedonists’ (*n* = 1109)	Cluster 3 ‘High-Preferring’ (*n* = 1040)	*p* **
I usually avoid eating fried foods	True	111 (41.1%)	400 (36.1%)	283 (27.2%)	<0.001
	False	159 (58.9%)	709 (63.9%)	757 (72.8%)	
I try to keep my overall fat intake down	True	155 (57.4%)	599 (54.0%)	529 (50.9%)	0.107
	False	115 (42.6%)	510 (46.0%)	511 (49.1%)	
I try to keep my overall sugar intake down	True	150 (55.5%)	655 (59.1%)	601 (57.8%)	0.554
	False	120 (44.5%)	454 (40.9%)	439 (42.2%)	
If I am having a dessert at home, I try to have something low in fat	True	88 (32.6%)	340 (30.7%)	279 (26.8%)	<0.001
	False	119 (44.1%)	617 (55.6%)	652 (62.7%)	
	Not applicable	63 (23.3%)	152 (13.7%)	109 (10.5%)	
I usually eat at least one serving of vegetables (excluding potatoes) or salad with my evening meal	True	175 (64.8%)	809 (72.9%)	815 (78.4%)	<0.001
	False	95 (35.2%)	300 (27.1%)	225 (21.6%)	
When I put butter or margarine on bread, I usually spread it thinly	True	154 (57.0%)	742 (66.9%)	660 (63.5%)	<0.001
	False	58 (21.5%)	198 (17.8%)	266 (25.6%)	
	Not applicable	58 (21.5%)	169 (15.2%)	114 (11.0%)	
If I have a packed lunch, I usually include some chocolate and/or biscuits	True	110 (40.7%)	483 (43.6%)	481 (46.2%)	<0.001
	False	61 (22.6%)	167 (15.1%)	206 (19.8%)	
	Not applicable	99 (36.7%)	459 (41.4%)	353 (33.9%)	
I often have cream on desserts	True	145 (53.7%)	722 (65.1%)	672 (64.6%)	<0.001
	False	45 (16.7%)	183 (16.5%)	206 (19.8%)	
	Not applicable	80 (29.6%)	204 (18.4%)	162 (15.6%)	

* AFHC—Adolescents’ Food Habits Checklist; ** chi^2^.

**Table 5:** The declared food consumption habits assessed based on the Adolescents’ Food Habits Checklist (AFHC) in the clusters stratified based on the preferences assessed while using Food Preference Questionnaire (FPQ) within the population of the second phase of the Polish Adolescents’ COVID-19 Experience (PLACE-19) Study (*n* = 2419).

Declared Food Consumption Habits Based on AFHC *		Cluster 1 ‘Low-Preferring’ (*n* = 270)	Cluster 2 ‘Hedonists’ (*n* = 1109)	Cluster 3 ‘High-Preferring’ (*n* = 1040)	p **
I usually eat a dessert or pudding if there is one available	True	113 (41.9%)	388 (35.0%)	245 (23.6%)	<0.001
	False	157 (58.1%)	721 (65.0%)	795 (76.4%)	
I make sure I eat at least one serving of fruit a day	True	171 (63.3%)	793 (71.5%)	789 (75.9%)	<0.001
	False	99 (36.7%)	316 (28.5%)	251 (24.1%)	
I avoid eating lots of sausages and burgers	True	127 (47.0%)	601 (54.2%)	539 (51.8%)	<0.001
	False	95 (35.2%)	410 (37.0%)	413 (39.7%)	
	Not applicable	48 (17.8%)	98 (8.8%)	88 (8.5%)	
I make sure I eat at least one serving of vegetables or salad a day	True	159 (58.9%)	754 (68.0%)	767 (73.8%)	<0.001
	False	111 (41.1%)	355 (32.0%)	273 (26.2%)	
I try to ensure I eat plenty of fruit and vegetables	True	173 (64.1%)	750 (67.6%)	802 (77.1%)	<0.001
	False	97 (35.9%)	359 (32.4%)	238 (22.9%)	
I often eat sweet snacks between meals	True	167 (61.9%)	645 (58.2%)	549 (52.8%)	0.006
	False	103 (38.1%)	464 (41.8%)	491 (47.2%)	
When I have a snack between meals, I often choose fruit	True	120 (44.4%)	648 (58.4%)	598 (57.5%)	<0.001
	False	83 (30.7%)	297 (26.8%)	326 (31.3%)	
	Not applicable	67 (24.8%)	164 (14.8%)	116 (11.1%)	
I eat at least three servings of fruit most days	True	122 (45.2%)	446 (40.2%)	460 (44.2%)	0.109
	False	148 (54.8%)	663 (59.8%)	580 (55.8%)	
I generally try to have a healthy diet	True	181 (67.0%)	790 (71.2%)	779 (74.9%)	0.019
	False	89 (33.0%)	319 (28.8%)	261 (25.1%)	

* AFHC—Adolescents’ Food Habits Checklist; ** chi^2^.

**Table 6:** The declared food purchase habits assessed based on the Adolescents’ Food Habits Checklist (AFHC) in the clusters stratified based on the preferences assessed while using the Food Preference Questionnaire (FPQ) within the female population of the second phase of the Polish Adolescents’ COVID-19 Experience (PLACE-19) Study (*n* = 1535).

Declared Food Purchase Habits Based on AFHC *		Cluster 1 ‘Low-Preferring’ (*n* = 174)	Cluster 2 ‘Hedonists’ (*n* = 747)	Cluster 3 ‘High-Preferring’ (*n* = 614)	*p* **
If I am having lunch away from home, I often choose a low-fat option	True	49 (28.2%)	189 (25.3%)	163 (26.5%)	0.080
	False	50 (28.7%)	194 (26.0%)	195 (31.8%)	
	Not applicable	75 (43.1%)	364 (48.7%)	256 (41.7%)	
If I am buying crisps, I often choose a low-fat brand	True	39 (22.4%)	138 (18.5%)	112 (18.2%)	0.015
	False	82 (47.1%)	407 (54.5%)	370 (60.3%)	
	Not applicable	53 (30.5%)	202 (27.0%)	132 (21.5%)	
I often buy pastries or cakes	True	132 (75.9%)	629 (84.2%)	501 (81.6%)	0.030
	False	42 (24.1%)	118 (15.8%)	113 (18.4%)	
I rarely eat takeaway meals	True	135 (77.6%)	652 (87.3%)	541 (88.1%)	0.001
	False	39 (22.4%)	95 (12.7%)	73 (11.9%)	
When I am buying a soft drink, I usually choose a diet drink	True	75 (43.1%)	340 (45.5%)	243 (39.6%)	0.088
	False	99 (56.9%)	407 (54.5%)	371 (60.4%)	
If I am having a dessert or pudding in a restaurant, I usually choose the healthiest one	True	38 (21.8%)	121 (16.2%)	102 (16.6%)	0.005
	False	62 (35.6%)	278 (37.2%)	278 (45.3%)	
	Not applicable	74 (42.5%)	348 (46.6%)	234 (38.1%)	

* AFHC—Adolescents’ Food Habits Checklist; ** chi^2^.

**Table 7:** The declared food preparation habits assessed based on the Adolescents’ Food Habits Checklist (AFHC) in the clusters stratified based on the preferences assessed while using the Food Preference Questionnaire (FPQ) within the female population of the second phase of the Polish Adolescents’ COVID-19 Experience (PLACE-19) Study (*n* = 1535).

Declared Food Preparation Habits Based on AFHC *		Cluster 1 ‘Low-Preferring’ (*n* = 174)	Cluster 2 ‘Hedonists’ (*n* = 747)	Cluster 3 ‘High-Preferring’ (*n* = 614)	*p* **
I usually avoid eating fried foods	True	88 (50.6%)	316 (42.3%)	207 (33.7%)	<0.001
	False	86 (49.4%)	431 (57.7%)	407 (66.3%)	
I try to keep my overall fat intake down	True	111 (63.8%)	453 (60.6%)	352 (57.3%)	0.232
	False	63 (36.2%)	294 (39.4%)	262 (42.7%)	
I try to keep my overall sugar intake down	True	114 (65.5%)	489 (65.5%)	400 (65.1%)	0.9912
	False	60 (34.5%)	258 (34.5%)	214 (34.9%)	
If I am having a dessert at home, I try to have something low in fat	True	60 (34.5%)	266 (35.6%)	191 (31.1%)	<0.001
	False	75 (43.1%)	382 (51.1%)	362 (59.0%)	
	Not applicable	39 (22.4%)	99 (13.3%)	61 (9.9%)	
I usually eat at least one serving of vegetables (excluding potatoes) or salad with my evening meal	True	126 (72.4%)	573 (76.7%)	507 (82.6%)	0.004
	False	48 (27.6%)	174 (23.3%)	107 (17.4%)	
When I put butter or margarine on bread, I usually spread it thinly	True	112 (64.4%)	519 (69.5%)	425 (69.2%)	0.001
	False	27 (15.5%)	117 (15.7%)	129 (21.0%)	
	Not applicable	35 (20.1%)	111 (14.9%)	60 (9.8%)	
If I have a packed lunch, I usually include some chocolate and/or biscuits	True	65 (37.4%)	302 (40.4%)	276 (45.0%)	<0.001
	False	44 (25.3%)	102 (13.7%)	115 (18.7%)	
	Not applicable	65 (37.4%)	343 (45.9%)	223 (36.3%)	
I often have cream on desserts	True	101 (58.0%)	498 (66.7%)	421 (68.6%)	0.001
	False	25 (14.4%)	121 (16.2%)	110 (17.9%)	
	Not applicable	48 (27.6%)	128 (17.1%)	83 (13.5%)	

* AFHC—Adolescents’ Food Habits Checklist; ** chi^2^.

**Table 8:** The declared food consumption habits assessed based on the Adolescents’ Food Habits Checklist (AFHC) in the clusters stratified based on the preferences assessed while using the Food Preference Questionnaire (FPQ) within the female population of the second phase of the Polish Adolescents’ COVID-19 Experience (PLACE-19) Study (*n* = 1535).

Declared Food Consumption Habits based on AFHC *		Cluster 1 ‘Low-Preferring’ (*n* = 174)	Cluster 2 ‘Hedonists’ (*n* = 747)	Cluster 3 ‘High-Preferring’ (*n* = 614)	p **
I usually eat a dessert or pudding if there is one available	True	76 (43.7%)	282 (37.8%)	163 (26.5%)	<0.001
	False	98 (56.3%)	465 (62.2%)	451 (73.5%)	
I make sure I eat at least one serving of fruit a day	True	125 (71.8%)	577 (77.2%)	501 (81.6%)	0.013
	False	49 (28.2%)	170 (22.8%)	113 (18.4%)	
I avoid eating lots of sausages and burgers	True	91 (52.3%)	438 (58.6%)	394 (64.2%)	<0.001
	False	56 (32.2%)	259 (34.7%)	177 (28.8%)	
	Not applicable	27 (15.5%)	50 (6.7%)	43 (7.0%)	
I make sure I eat at least one serving of vegetables or salad a day	True	118 (67.8%)	552 (73.9%)	499 (81.3%)	<0.001
	False	56 (32.2%)	195 (26.1%)	115 (18.7%)	
I try to ensure I eat plenty of fruit and vegetables	True	128 (73.6%)	567 (75.9%)	527 (85.8%)	<0.001
	False	46 (26.4%)	180 (24.1%)	87 (14.2%)	
I often eat sweet snacks between meals	True	107 (61.5%)	432 (57.8%)	337 (54.9%)	0.251
	False	67 (38.5%)	315 (42.2%)	277 (45.1%)	
When I have a snack between meals, I often choose fruit	True	92 (52.9%)	496 (66.4%)	392 (63.8%)	<0.001
	False	38 (21.8%)	122 (16.3%)	153 (24.9%)	
	Not applicable	44 (25.3%)	129 (17.3%)	69 (11.2%)	
I eat at least three servings of fruit most days	True	92 (52.9%)	333 (44.6%)	292 (47.6%)	0.123
	False	82 (47.1%)	414 (55.4%)	322 (52.4%)	
I generally try to have a healthy diet	True	127 (73.0%)	564 (75.5%)	492 (80.1%)	0.052
	False	47 (27.0%)	183 (24.5%)	122 (19.9%)	

* AFHC—Adolescents’ Food Habits Checklist; ** chi^2^.

**Table 9:** The declared food purchase habits assessed based on the Adolescents’ Food Habits Checklist (AFHC) in the clusters stratified based on the preferences assessed while using the Food Preference Questionnaire (FPQ) within the male population of the second phase of the Polish Adolescents’ COVID-19 Experience (PLACE-19) Study (*n* = 884).

Declared Food Purchase Habits Based on AFHC *		Cluster 1 ‘Low-Preferring’ (*n* = 96)	Cluster 2 ‘Hedonists’ (*n* = 362)	Cluster 3 ‘High-Preferring’ (*n* = 426)	*p* **
If I am having lunch away from home, I often choose a low-fat option	True	30 (31.3%)	74 (20.4%)	90 (21.1%)	0.043
	False	33 (34.4%)	163 (45.0%)	210 (49.3%)	
	Not applicable	33 (34.4%)	125 (34.5%)	126 (29.6%)	
If I am buying crisps, I often choose a low-fat brand	True	18 (18.8%)	52 (14.4%)	63 (14.8%)	0.158
	False	48 (50.0%)	227 (62.7%)	271 (63.6%)	
	Not applicable	30 (31.3%)	83 (22.9%)	92 (21.6%)	
I often buy pastries or cakes	True	68 (70.8%)	275 (76.0%)	287 (67.4%)	0.029
	False	28 (29.2%)	87 (24.0%)	139 (32.6%)	
I rarely eat takeaway meals	True	61 (63.5%)	274 (75.7%)	323 (75.8%)	0.035
	False	35 (36.5%)	88 (24.3%)	103 (24.2%)	
When I am buying a soft drink, I usually choose a diet drink	True	38 (39.6%)	117 (32.3%)	118 (27.7%)	0.056
	False	58 (60.4%)	245 (67.7%)	308 (72.3%)	
If I am having a dessert or pudding in a restaurant, I usually choose the healthiest one	True	14 (14.6%)	44 (12.2%)	50 (11.7%)	0.963
	False	51 (53.1%)	199 (55.0%)	236 (55.4%)	
	Not applicable	31 (32.3%)	119 (32.9%)	140 (32.9%)	

* AFHC—Adolescents’ Food Habits Checklist; ** chi^2^.

**Table 10:** The declared food preparation habits assessed based on the Adolescents’ Food Habits Checklist (AFHC) in the clusters stratified based on the preferences assessed while using the Food Preference Questionnaire (FPQ) within the male population of the second phase of the Polish Adolescents’ COVID-19 Experience (PLACE-19) Study (*n* = 884).

Declared Food Preparation Habits Based on AFHC *		Cluster 1 ‘Low-Preferring’ (*n* = 96)	Cluster 2 ‘Hedonists’ (*n* = 362)	Cluster 3 ‘High-Preferring’ (*n* = 426)	*p* **
I usually avoid eating fried foods	True	23 (24.0%)	84 (23.2%)	76 (17.8%)	0.127
	False	73 (76.0%)	278 (76.8%)	350 (82.2%)	
I try to keep my overall fat intake down	True	44 (45.8%)	146 (40.3%)	177 (41.5%)	<0.001
	False	52 (54.2%)	216 (59.7%)	249 (58.5%)	
I try to keep my overall sugar intake down	True	36 (37.5%)	166 (45.9%)	201 (47.2%)	0.226
	False	60 (62.5%)	196 (54.1%)	225 (52.8%)	
If I am having a dessert at home, I try to have something low in fat	True	28 (29.2%)	74 (20.4%)	88 (20.7%)	0.001
	False	44 (45.8%)	235 (64.9%)	290 (68.1%)	
	Not applicable	24 (25.0%)	53 (14.6%)	48 (11.3%)	
I usually eat at least one serving of vegetables (excluding potatoes) or salad with my evening meal	True	49 (51.0%)	236 (65.2%)	308 (72.3%)	<0.001
	False	47 (49.0%)	126 (34.8%)	118 (27.7%)	
When I put butter or margarine on bread, I usually spread it thinly	True	42 (43.8%)	223 (61.6%)	235 (55.2%)	0.001
	False	31 (32.3%)	81 (22.4%)	137 (32.2%)	
	Not applicable	23 (24.0%)	58 (16.0%)	54 (12.7%)	
If I have a packed lunch, I usually include some chocolate and/or biscuits	True	45 (46.9%)	181 (50.0%)	205 (48.1%)	0.701
	False	17 (17.7%)	65 (18.0%)	91 (21.4%)	
	Not applicable	34 (35.4%)	116 (32.0%)	130 (30.5%)	
I often have cream on desserts	True	44 (45.8%)	224 (61.9%)	251 (58.9%)	0.006
	False	20 (20.8%)	62 (17.1%)	96 (22.5%)	
	Not applicable	32 (33.3%)	76 (21.0%)	79 (18.5%)	

* AFHC—Adolescents’ Food Habits Checklist; ** chi^2^.

**Table 11:** The declared food consumption habits assessed based on the Adolescents’ Food Habits Checklist (AFHC) in the clusters stratified based on the preferences assessed while using the Food Preference Questionnaire (FPQ) within the male population of the second phase of the Polish Adolescents’ COVID-19 Experience (PLACE-19) Study (*n* = 884).

Declared Food Consumption Habits Based on AFHC *		Cluster 1 ‘Low-Preferring’ (*n* = 96)	Cluster 2 ‘Hedonists’ (*n* = 362)	Cluster 3 ‘High-Preferring’ (*n* = 426)	p **
I usually eat a dessert or pudding if there is one available	True	37 (38.5%)	106 (29.3%)	82 (19.2%)	<0.001
	False	59 (61.5%)	256 (70.7%)	344 (80.8%)	
I make sure I eat at least one serving of fruit a day	True	46 (47.9%)	216 (59.7%)	288 (67.6%)	0.001
	False	50 (52.1%)	146 (40.3%)	138 (32.4%)	
I avoid eating lots of sausages and burgers	True	36 (37.5%)	163 (45.0%)	145 (34.0%)	<0.001
	False	39 (40.6%)	151 (41.7%)	236 (55.4%)	
	Not applicable	21 (21.9%)	48 (13.3%)	45 (10.6%)	
I make sure I eat at least one serving of vegetables or salad a day	True	41 (42.7%)	202 (55.8%)	268 (62.9%)	0.001
	False	55 (57.3%)	160 (44.2%)	158 (37.1%)	
I try to ensure I eat plenty of fruit and vegetables	True	45 (46.9%)	183 (50.6%)	275 (64.6%)	<0.001
	False	51 (53.1%)	179 (49.4%)	151 (35.4%)	
I often eat sweet snacks between meals	True	60 (62.5%)	213 (58.8%)	212 (49.8%)	0.011
	False	36 (37.5%)	149 (41.2%)	214 (50.2%)	
When I have a snack between meals, I often choose fruit	True	28 (29.2%)	152 (42.0%)	206 (48.4%)	<0.001
	False	45 (46.9%)	175 (48.3%)	173 (40.6%)	
	Not applicable	23 (24.0%)	35 (9.7%)	47 (11.0%)	
I eat at least three servings of fruit most days	True	30 (31.3%)	113 (31.2%)	168 (39.4%)	0.038
	False	66 (68.8%)	249 (68.8%)	258 (60.6%)	
I generally try to have a healthy diet	True	54 (56.3%)	226 (62.4%)	287 (67.4%)	0.083
	False	42 (43.8%)	136 (37.6%)	139 (32.6%)	

* AFHC—Adolescents’ Food Habits Checklist; ** chi^2^.

## Supplementary Materials

The following are available online at https://www.mdpi.com/article/10.3390/nu13093003/s1, Supplementary Table S1. The summary of the comparison of the declared food habits assessed based on Adolescents’ Food Habits Checklist (AFHC) in the clusters stratified based on the preferences assessed while using Food Preference Questionnaire (FPQ) within the population of the second phase of the Polish Adolescents’ COVID-19 Experience (PLACE-19) Study for female and male respondents.
