# Supplementary material for: Association between Food Preferences and Food Habits in a Polish Adolescents’ COVID-19 Experience (PLACE-19) Study
Source: Nutrients. 2021 Aug 28;13(9):3003. doi: 10.3390/nu13093003 (PMC8471474; doi:10.3390/nu13093003)
Supplement: Supplementary file 1 [file nutrients-13-03003-s001.zip › nutrients-1351037-supplementary.pdf]

nutrients

Supplementary Material

Analysis of Gender-Dependent Association between Food Preferences and Food Habits in a Polish Adolescents' COVID-19 Experience (PLACE-19) Study

Dominika Skolmowska [1], Dominika Głąbska [1,*] and Dominika Guzek [2]

Supplementary Table S1. The summary of the comparison of the declared food habits assessed based on Adolescents' Food Habits Checklist (AFHC) in the clusters stratified based on the preferences assessed while using Food Preference Questionnaire (FPQ) within the population of the second phase of the Polish Adolescents' COVID-19 Experience (PLACE-19) Study for female and male respondents.

Declared Food Habits Based on AFHC *		Difference of Declared Frequency of Habit between Clusters	
		Female	Male
Food purchase habits	If I am having lunch away from home, I often choose a low-fat option	ns	↑ 'low-preferring'
	If I am buying crisps, I often choose a low-fat brand	↑ 'low-preferring'	ns
	I often buy pastries or cakes	↓ 'low-preferring'	↑ 'hedonists'
	I rarely eat takeaway meals	↓ 'low-preferring'	↓ 'low-preferring'
	When I am buying a soft drink, I usually choose a diet drink	ns	ns
	If I am having a dessert or pudding in a restaurant, I usually choose the healthiest one	↑ 'low-preferring'	ns
Food preparation habits	I usually avoid eating fried foods	↑ 'low-preferring' ↓ 'high-preferring'	ns
	I try to keep my overall fat intake down	ns	↑ 'low-preferring'
	I try to keep my overall sugar intake down	ns	ns
	If I am having a dessert at home, I try to have something low in fat	↓ 'high-preferring'	↑ 'low-preferring'
	I usually eat at least one serving of vegetables (excluding potatoes) or salad with my evening meal	↑ 'high-preferring'	↓ 'low-preferring'
	When I put butter or margarine on bread, I usually spread it thinly	↓ 'low-preferring'	↓ 'low-preferring'
	If I have a packed lunch, I usually include some chocolate and/or biscuits	↑ 'high-preferring'	ns
	I often have cream on desserts	↓ 'low-preferring'	↓ 'low-preferring'
Food consumption habits	I usually eat a dessert or pudding if there is one available	↓ 'high-preferring'	↓ 'high-preferring'
	I make sure I eat at least one serving of fruit a day	↓ 'low-preferring'	↓ 'low-preferring'
	I avoid eating lots of sausages and burgers	↓ 'low-preferring'	↑ 'hedonists'
	I make sure I eat at least one serving of vegetables or salad a day	↑ 'high-preferring'	↓ 'low-preferring'
	I try to ensure I eat plenty of fruit and vegetables	↑ 'high-preferring'	↑ 'high-preferring'
	I often eat sweet snacks between meals	ns	↓ 'high-preferring'
	When I have a snack between meals, I often choose fruit	↓ 'low-preferring'	↓ 'low-preferring'
	I eat at least three servings of fruit most days	ns	↑ 'high-preferring'
	I generally try to have a healthy diet	ns	ns

* AFHC–Adolescents' Food Habits Checklist; ns–not significant.
